# Novel insights into the association between genetically proxied inhibition of proprotein convertase subtilisin/kexin type 9 and risk of sarcopenia

**DOI:** 10.1002/jcsm.13575

**Published:** 2024-09-10

**Authors:** Hongyan Jiang, Lulu Li, Xue Zhang, Jia He, Chuanhuai Chen, Ruimin Sun, Ying Chen, Lijuan Xia, Lei Wen, Yunxiang Chen, Junxiu Liu, Lijiang Zhang, Wanqiang Lv

**Affiliations:** ^1^ Center of Safety Evaluation and Research, Key Laboratory of Drug Safety Evaluation and Research of Zhejiang Province Hangzhou Medical College Zhejiang China; ^2^ Department of Emergency Medicine, The First Affiliated Hospital, School of Medicine Zhejiang University Zhejiang China

**Keywords:** Mendelian randomization, Older people, Proprotein convertase subtilisin/kexin type 9, Sarcopenia, Therapeutic target prediction

## Abstract

**Background:**

The effects of lipid‐lowering drugs [including statins, ezetimibe, and proprotein convertase subtilisin/kexin type 9 (PCSK9) inhibitors] on hyperlipidaemia have been established. Some may have treatment effects beyond their reported properties, offering potential opportunities for drug repurposing. Epidemiological studies have reported conflicting findings on the relationship between lipid‐lowering medication use and sarcopenia risk.

**Methods:**

We performed a two‐sample Mendelian randomization (MR) study to investigate the causal association between the use of genetically proxied lipid‐lowering drugs (including statins, ezetimibe, and PCSK9 inhibitors, which use low‐density lipoprotein as a biomarker), and sarcopenia risk. The inverse‐variance weighting method was used with pleiotropy‐robust methods (MR–Egger regression and weighted median) and colocalization as sensitivity analyses.

**Results:**

According to the positive control analysis, genetically proxied inhibition in lipid‐lowering drug targets was associated with a lower risk of coronary heart disease [PCSK9 (OR, 0.67; 95% CI, 0.61 to 0.72; *P =* 7.7E‐21); 3‐hydroxy‐3‐methylglutaryl coenzyme A reductase (HMGCR; OR, 0.68; 95% CI, 0.57 to 0.82; *P =* 4.6E‐05), and Niemann–Pick C1‐like 1 (NPC1L1; OR, 0.53; 95% CI, 0.40 to 0.69; *P =* 3.3E‐06)], consistent with drug mechanistic actions and previous trial evidence. Genetically proxied inhibition of PCSK9 (beta, −0.040; 95% CI, −0.068 to −0.012; *P =* 0.005) and circulating PCSK9 levels (beta, −0.019; 95% CI, −0.033 to −0.005; *P =* 0.006) were associated with reduced appendicular lean mass (ALM) with concordant estimates in terms of direction and magnitude. Validation analyses using a second instrument for PCSK9 yielded consistent results in terms of direction and magnitude [(PCSK9 to ALM; beta, −0.052; 95% CI, −0.074 to −0.032; *P =* 7.1E‐7); (PCSK9 protein to ALM; beta, −0.060; 95% CI, −0.106 to −0.014; *P =* 0.010)]. Genetically proxied inhibition of PCSK9 gene expression in the liver may be associated with reduced ALM (beta, −0.013; 95% CI, −0.035 to 0.009; *P =* 0.25), consistent with the results of PCSK9 drug‐target and PCSK9 protein MR analyses, but the magnitude was less precise. No robust association was found between HMGCR inhibition (beta, 0.048; 95% CI, −0.015 to 0.110; *P =* 0.14) or NPC1L1 (beta, 0.035; 95% CI, −0.074 to 0.144; *P =* 0.53) inhibition and ALM, and validation and sensitivity MR analyses showed consistent estimates.

**Conclusions:**

This MR study suggested that PCSK9 is involved in sarcopenia pathogenesis and that its inhibition is associated with reduced ALM. These findings potentially pave the way for future studies that may allow personalized selection of lipid‐lowering drugs for those at risk of sarcopenia.

## Introduction

Sarcopenia has been classified as a muscle disease according to the International Classification of Disease (ICD‐10: M62.84) and is associated with adverse outcomes, including functional decline and frailty.^1^ There is no established pharmacological approach for treating sarcopenia, highlighting the unmet need for preventive and therapeutic treatments; however, developing drugs for the treatment of sarcopenia is very challenging.^2^ One alternative approach is drug repurposing, which involves the use of licenced drugs with well‐established safety and pharmacokinetic profiles for new indications.^3^ Drug repositioning is suitable for the urgent need for clinical treatment of sarcopenia, and recent studies have provided an approach to address this issue.^4^, ^5^

Mendelian randomization (MR) uses genetic variants as instrumental variables (IVs) for exposure to provide the unconfounded effect of the exposure on the outcome,^6^ which is a viable approach to gauge the repurposing potential of a drug. With the increasing availability of genome‐wide association study (GWAS) data, it is becoming increasingly feasible to study drug effects by investigating genetic variants in the genes of their protein targets. Genetic variants from the gene locus that encode the drug target protein are likely to influence the expression or function of the protein. Leveraging these variants as genetic instruments can mimic how the drug modulates its target protein, allowing us to estimate the effect of genetic variation in the drug target on a new indication, such as a randomized controlled trial.

We focused on lipid abnormalities in this study, due to their established role in metabolic and cardiovascular diseases and emerging evidence suggesting their impact on muscle health and sarcopenia. Low‐density lipoprotein (LDL) cholesterol‐lowering drugs commonly prescribed for the prevention and management of cardiovascular disease, including 3‐Hydroxy‐3‐methylglutaryl coenzyme A reductase inhibitors (HMGCR; target of statins), Niemann‐Pick C1‐Like 1 (NPC1L1; target of ezetimibe) and proprotein convertase subtilisin/kexin type 9 (PCSK9; target of PCSK9 inhibitors). The effects of lipid‐lowering drugs on hyperlipidaemia have been established through many outcome‐based RCTs, and these drugs have been found to be effective and safe. Some drugs may have treatment effects beyond their reported properties,^4^ offering potential opportunities for drug repurposing. However, the observational studies used to explore such opportunities are impacted by common biases, including confounding by indication.^6^ Given these aspects, genetic variants in genes corresponding to the targets of three lipid‐lowering drugs served as a proxy for the effects of pharmacological treatments, and we aimed to evaluate the repurposing of commonly used lipid‐lowering drugs for the treatment of sarcopenia in an MR framework.

## Materials and methods

### Study design

**Figure 1:**
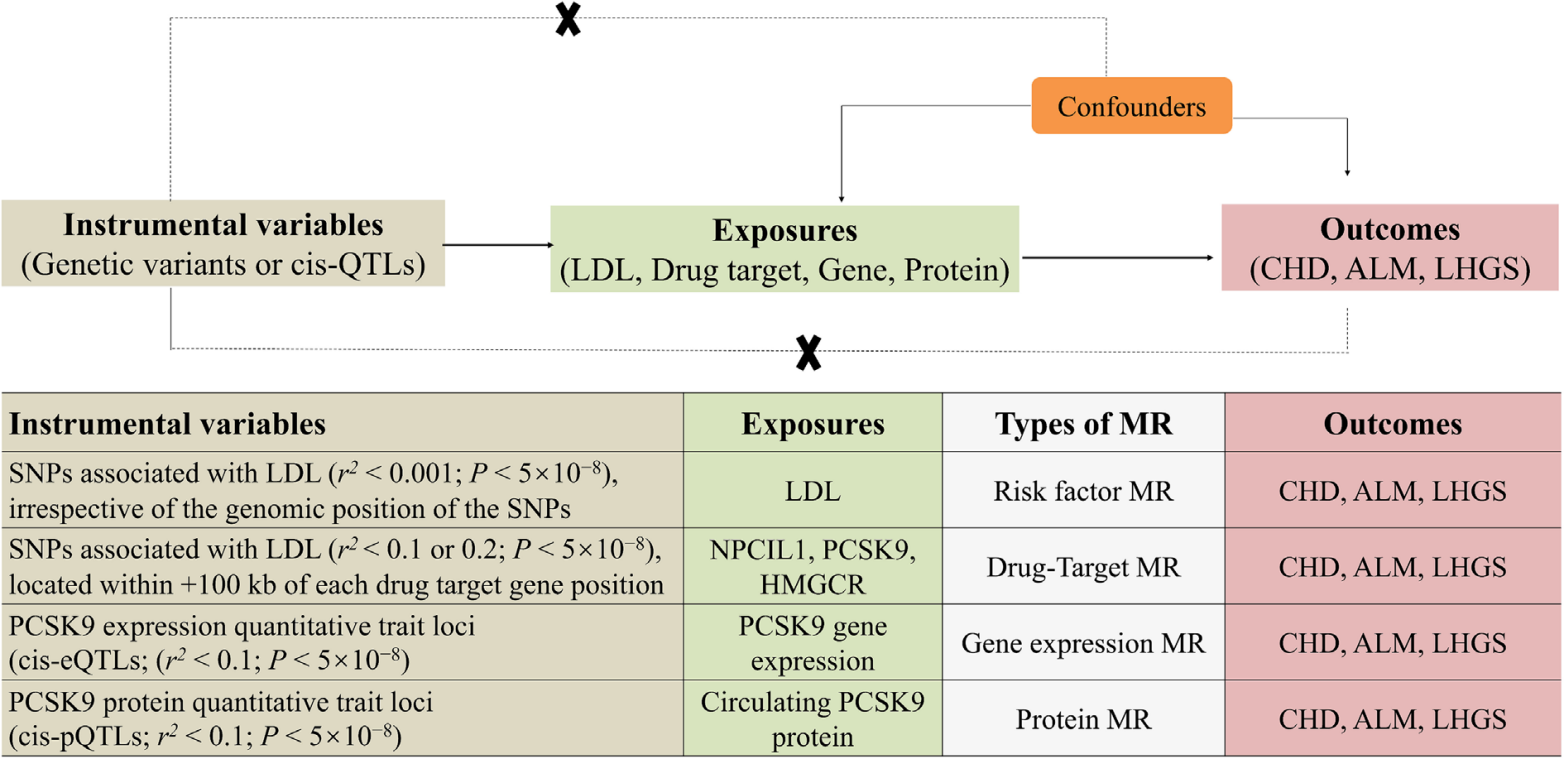
Study design overview. (A) MR model. (B) Genetic instrument construction and analysis plan in the present study. For each drug target or LDL, genetic instruments were constructed by obtaining summary genetic association data on SNPs associated with LDL located within or near the gene encoding the drug target ([HMGCR; chr5:74,632,154‐74,657,929], [NPC1L1; chr7:44,552,134‐44,580,914], [PCSK9; chr1:55,505,221‐55,530,525]) or independent of genomic position (LDL) from the Global Lipids Genetics Consortium (GLGC). We constructed instruments for circulating PCSK9 levels from pQTLs in participants in the deCODE cohort (*n* = 35,559). We constructed a second PCSK9 protein instrument derived from a separate study providing PCSK9 pQTLs in 12,271 participants. Liver PCSK9 expression data were derived from genotype‐tissue expression version 8 liver tissue (*n* = 178). Cis‐located variants (+/− 100 kb) extracted from respective pQTL and eQTL GWASs; summary genetic association data for these SNPS were then extracted from genome‐wide association studies of CHD, ALM and LHGS. MR analyses were performed using inverse‐variance weighted random‐effects models as primary analyses and various approaches as sensitivity analyses. ALM, appendicular lean mass; CHD, coronary heart disease; HMGCR, 3‐hydroxy‐3‐methylglutaryl‐CoA reductase; LDL, low‐density lipoprotein; LHGS, low handgrip strength; MR, Mendelian randomization; NPC1LI, Niemann–Pick C1‐like 1; PCSK9, proprotein convertase subtilisin/kexin type 9.

Figure 1 depicts the diagram of the present MR study. First, we searched the ChEMBL database to identify the possible drug classes for hyperlipidaemia and then utilized the DrugBank database to identify target genes associated with each drug class. Second, we identified genetic instruments to proxy drug classes. The genetic instruments used to proxy lipid‐lowering drugs were extracted from previously published GWASs of LDL. Third, we conducted an MR analysis of the associations between those genetic instruments and positive controls to assess the validity of those genetic instruments, given the recognized benefits of all three drugs in this context. Genetically proxied drugs not associated with the positive controls were excluded. The final step involved assessing the effects of the selected genetic instruments on sarcopenia risk.

This study used publicly available, deidentified summary data from prior GWASs. Details about all the GWASs used in our study are listed in Table 1. All cohort data included in the GWAS used in the present study were obtained with individual approval from relevant ethical review boards.

**Table 1:** Summary‐level data sets of GWAS used in the MR analyses

Exposure/outcome	Traits	Cohort	Participants	Web source
Exposures	Low‐density lipoprotein (LDL)	GLGC	1 320 016 individuals	http://csg.sph.umich.edu/willer/public/glgc‐lipids2021/results/ancestry_specific/
	Low‐density lipoprotein (LDL)	GLGC	149 555 individuals	https://csg.sph.umich.edu/willer/public/lipids2013/
	PCSK9 cis‐pQTLs	deCODE	35 362 individuals	https://www.decode.com/summarydata/
	PCSK9 cis‐pQTLs replication	LIFE‐Heart, LIFE‐Adult, LURIC, CAP, and TwinGene	12 271 individuals	https://doi.org/10.5281/zenodo.5643551.
	PCSK9 liver eQTLs	GTEx	178 individuals	https://gtexportal.org/home/datasets
Outcomes	Coronary heart disease (CHD)	CARDIoGRAMplusC4D	60 801 cases and 123 504 controls	http://www.cardiogramplusc4d.org
	Appendicular lean mass (ALM; UK Biobank)	UK Biobank	450 243 participants	UK Biobank https://www.leelabsg.org/resources
	Low handgrip strength (LHGS; FNIH)	The CHARGE consortium	20 335 cases and 236 188 controls	www.mskkp.org
	Low handgrip strength (LHGS; EWGSOP)	The CHARGE consortium	48 596 cases and 207 927 controls	www.mskkp.org

### Genetic variant selection for LDL

Genetic variants associated with LDL were taken from two publications by the Global Lipids Genetics Consortium (GLGC).^7^, ^8^ In the present study, the primary data source was the largest GWAS meta‐analysis to date by GLGC, comprising approximately 1.3 million individuals of European ancestry.^7^ Data excluding UK Biobank (*n* = 842 660) or Finnish (*n* = 1 177 987) participants were used for our analyses, using outcomes from the UK Biobank to reduce potential bias from sample overlap. The other data source was the most cited GWAS meta‐analysis for blood lipid levels, consisting primarily of individuals of European ancestry.^8^ To proxy for genetically determined LDL, single‐nucleotide polymorphisms (SNPs) associated with LDL (*r* ^2^ < 0.001; *P* < 5 × 10^−8^) were selected as IVs, irrespective of the genomic position of the SNPs. Finally, the selected genetic proxies of LDL that were acquired from those two sources are presented in Tables S1 and S2.

### Genetic variant selection for lipid‐lowering drugs

We identified the gene(s) that encode the target protein(s) of current LDL‐lowering drugs from the DrugBank and ChEMBL databases, choosing independent variants from the gene locus as genetic instruments to proxy the pharmacological modulation of the drug target protein. Genes encoding the target proteins of these drugs were identified from the DrugBank (v5.0) and ChEMBL (v29.0) databases and are presented in Table S3. In summary, the molecular targets of current LDL‐lowering drugs include HMGCR (a statin target), NPC1L1 (an ezetimibe target) and PCSK9 (a PCSK9 inhibitor target). We chose LDL as the biomarker because each of these drugs has been shown to reduce LDL levels.

Because a reduction in LDL is the primary biomarker measured to assess the physiological response to the three lipid‐lowering drugs,^9^ we used GWAS data on LDL levels.^7^, ^10^ To construct genetic instruments for the three lipid‐lowering drugs, genetic association estimates for LDL were acquired from the largest GWAS meta‐analysis on 1 320 016 individuals of European ancestry included in the GLGC.^7^ The variants with genome‐wide significance (*P* < 5 × 10^−8^) located within +500 kb of the drug target gene position (Build 37) were considered eligible instrumental variants. To maximize the strength of the instruments, the variants were limited to those with weak linkage disequilibrium (LD; *r* ^2^ < 0.1). Finally, 15 variants were selected to proxy LDL lowering through inhibition of HMGCR, 10 for NPC1L1, and 36 for PCSK9; the characteristics are presented in Table S4.

In causal inference analysis, validation analysis was often performed to enhance the reliability of the results, often conducted using instrumental variables with different linkage disequilibrium (LD). We constructed a second instrument for the three lipid‐lowering drugs for validation analysis. Genetic variants were extracted from the target gene region and its neighbouring ±100 kb window using the 1000 Genomes Phase 3 reference panel, and the genome‐wide significant SNPs were retained and filtered by a relaxed LD threshold with *r* ^2^ < 0.2. Genetic association estimates for LDL were acquired from the most cited GWAS meta‐analysis of LDL.^8^ A recent MR study by Yarmolinsky et al.^11^ identified the IVs for the three lipid‐lowering drugs with the same filtrations, and we selected the same sets of IVs for use in the present MR study. Briefly, five variants were selected to proxy LDL lowering through the inhibition of HMGCR, three for NPC1L1, and 11 for PCSK9, and their characteristics are presented in Table S5.

### Genetic variant selection for the PCSK9 quantitative trait locus

According to the results of drug‐target MR analyses, we found that the association between PCSK9 inhibition and ALM was considered “strong evidence”. Thus, we attempted to replicate the potential association between PCSK9 and genetic parameters, such as circulating protein levels (i.e., protein quantitative trait loci [pQTLs]) and gene expression data (i.e., expression quantitative trait loci [eQTLs]), on sarcopenia risk. Genome‐wide significant (*P* < 5 × 10^−8^) uncorrelated (r^2^ < 0.1) instrumental variants acting in cis (+100 kB from the PCSK9 gene region) were extracted for the determination of PCSK9 protein levels and PCSK9 gene expression.

We constructed instruments for circulating PCSK9 levels in participants from the deCODE cohort (*n* = 35 559),^12^ as presented in Table S6. We constructed a second PCSK9 protein instrument derived from a separate study providing PCSK9 pQTLs in 12 271 participants^13^ for validation analysis, as presented in Table S7. PCSK9 protein levels were measured in normalized protein units. PCSK9 expression data were derived from the genotype‐tissue expression (GTEx) Project summary data (Version 8, *n* = 266) in the liver (with knowledge and confirmed high PCSK9 expression in the liver),^14^, ^15^ and the instrumental variants for PCSK9 eQTLs are presented in Table S8. The eQTL data were measured in transcripts per million.^16^

### Genetic association data sources for sarcopenia

We selected grip strength as a measure of muscle strength and lean body mass as a measure of muscle mass, both of which are known to be valid trait predictors of sarcopenia.^17^ The GWAS summary data of low handgrip strength (LHGS) were obtained from the GWAS meta‐analysis of 256 523 Europeans aged 60 years and over.^18^ The data were summarized according to the LHGS criteria of the European Working Group on Sarcopenia in Older People (EWGSOP)^19^ (grip strength < 30 kg for males; <20 kg for females) or the Foundations of the National Institutes of Health (FNIH)^20^ (<26 kg for males and <16 kg for females). In the present study, we used GWAS data summarized according to two criteria for sarcopenia, LHGS (EWGSOP) and LHGS (FNIH).

There are no available GWAS data for muscle mass with cutoff values in the European population. We chose appendicular lean mass (ALM) as the outcome of sarcopenia. ALM accounts for ≥75% of skeletal muscle in the body and is also a widely studied indicator among sarcopenia patients.^21^ GWAS data were obtained from a meta‐analysis of 450 243 UK Biobank participants.^22^

### MR analysis and sensitivity analyses

MR analyses were performed using the R package TwoSampleMR.^23^ When two or more genetic instruments were available, inverse‐variance weighting MR (MR‐IVW) was applied, followed by heterogeneity analysis. MR–Egger regression and weighted median methods were used for sensitivity analyses. The Wald ratio was used if only one SNP was available for a given exposure. For the genetic instruments absent in the sarcopenia GWAS, we used the European panel from the 1000 Genomes Project Phase 3 as the reference panel and chose the SNPs in high LD (*r* ^2^  >  0.8) with the genetic instruments as a proxy. To examine whether genetic instruments affect sarcopenia risk through pathways other than the drug target of interest, we also searched for traits associated with these genetic instruments in the GWAS Catalogue. The *F* statistic was calculated to test the strength of the genetic instrument, and an *F* statistic above 10 typically indicates a strong instrument.

To account for multiple testing, Bonferroni correction was used to establish the threshold of *P* < 0.008 (0.05/6 [3 classes of drugs × 2 outcomes]), which we used as a heuristic to define ‘strong evidence’. The results with a *P* value ≥0.008 and a *P* value <0.05 were considered ‘suggestive evidence’.

### Positive control MR analysis

A positive control MR analysis serves to justify the genetic instruments of each drug target by demonstrating the expected effect on the outcome, which has an established causal relationship with the drug of interest. In this study, the SNPs that were selected as genetic instruments were validated by examining the association of genetically proxied drug target perturbations with endpoints shown to be influenced by these medications in RCTs. Given the recognized benefits of lipid‐lowering drugs, we examined the validity of genetic instruments by using coronary heart disease (CHD) as a positive control outcome. CHD summary statistics were taken from a GWAS of 60 801 clinically confirmed patients and 123 504 control participants.^24^

### Colocalization analysis

Bayesian colocalization analysis is used to assess the probability that two traits share the same causal variant.^25^ In the present study, we set the prior probability of the association between each variant with either trait to be 1 × 10^−4^ and the prior probability of a shared causal variant between two traits to be 1 × 10^−5^ using the “coloc” package (https://github.com/chrlswallace/coloc) with default arguments.^25^

## Results

### Genetic proxies for reductions in LDL in individuals with sarcopenia

Table S9 presents the causal estimates between LDL and outcomes [control outcome (CHD), ALM, LHGS (EWGSOP) and LHGS (FNIH)], comprising two sets of genetic instruments for LDL in MR analyses. The results of positive control MR analyses indicated that a genetically proxied inhibition in LDL was associated with a lower CHD risk, as expected, confirming the validity of the selected genetic instruments for LDL.

The results from the IVW MR analyses indicated that a genetically significant inhibition in LDL (due to genetic predisposition across 382 SNPs) may be associated with greater ALM (beta, 0.0293; 95% CI, 0.0005 to 0.0580; *P =* 0.046), but the MR sensitivity analyses did not show a consistent direction or magnitude. A secondary set of analyses using a set of 76 SNPs instead of a set of 382 SNPs yielded consistent results and provided less precise estimates. Taken together, these results suggest that the association between LDL and ALM requires further investigation. There was little statistical evidence of an association between LDL and LHGS in either set of genetic instruments.

### Genetically proxied classes of lipid‐lowering drugs and sarcopenia

**Figure 2:**
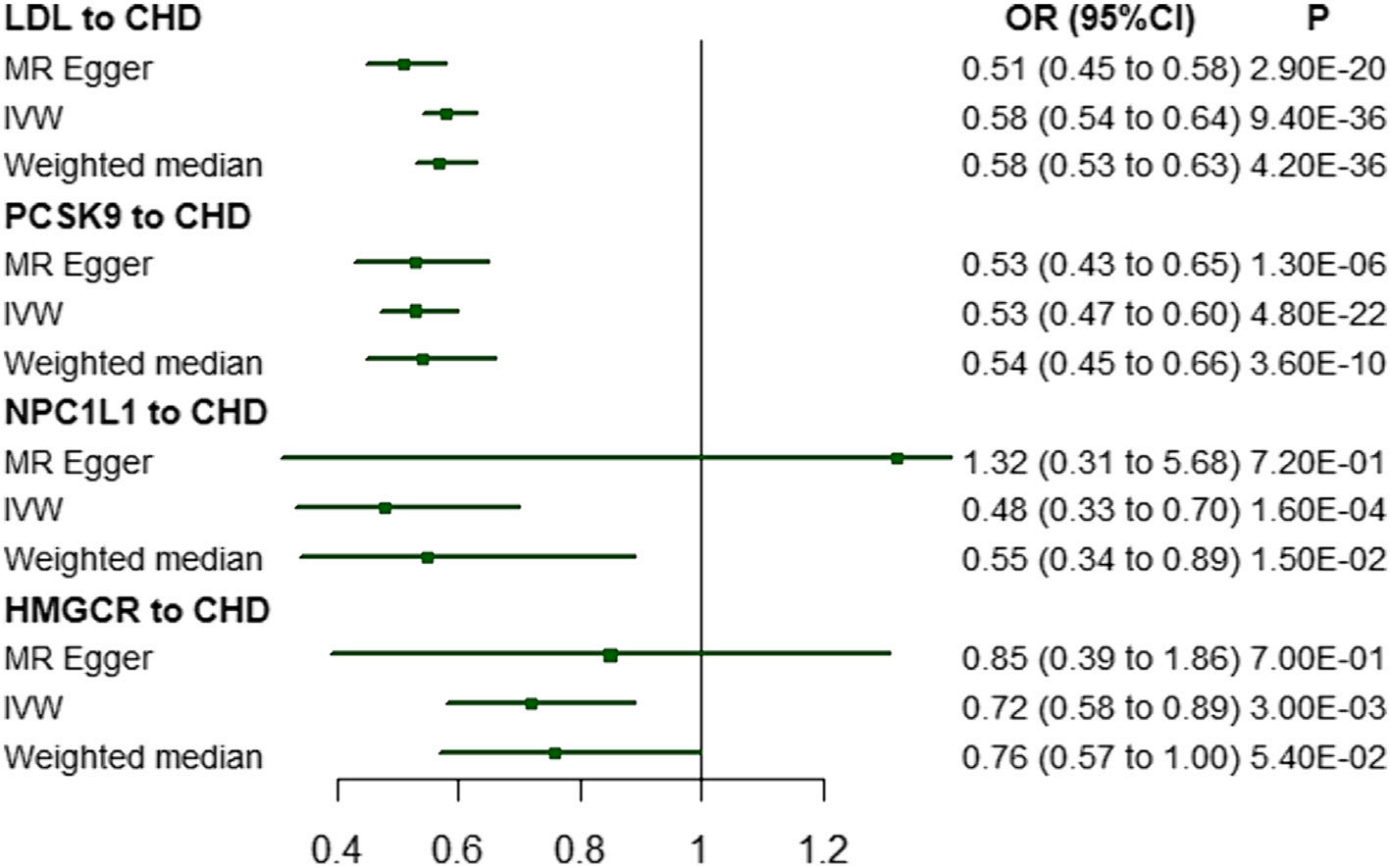
Associations between genetically proxied lipid‐lowering drugs, LDL and risk of coronary heart disease as the positive control in the primary analysis. CHD, coronary heart disease; CI, confidence interval; HMGCR, 3‐hydroxy‐3‐methylglutaryl CoA reductase; LDL, Iow‐density lipoprotein; NPCIL1, Niemann–Pick C1‐like 1; OR, odds ratio; PCSK9, proprotein convertase subtilisin/kexin type 9.

Table S10 presents the causal estimates between the genetically proxied inhibition of all three drug targets and the outcomes of two sets of genetic instruments. As expected, genetically proxied inhibition of all three drug targets was associated with a reduced risk of CHD according to two sets of genetic instruments, Figure 2 presents the results of the primary analysis.

**Figure 3:**
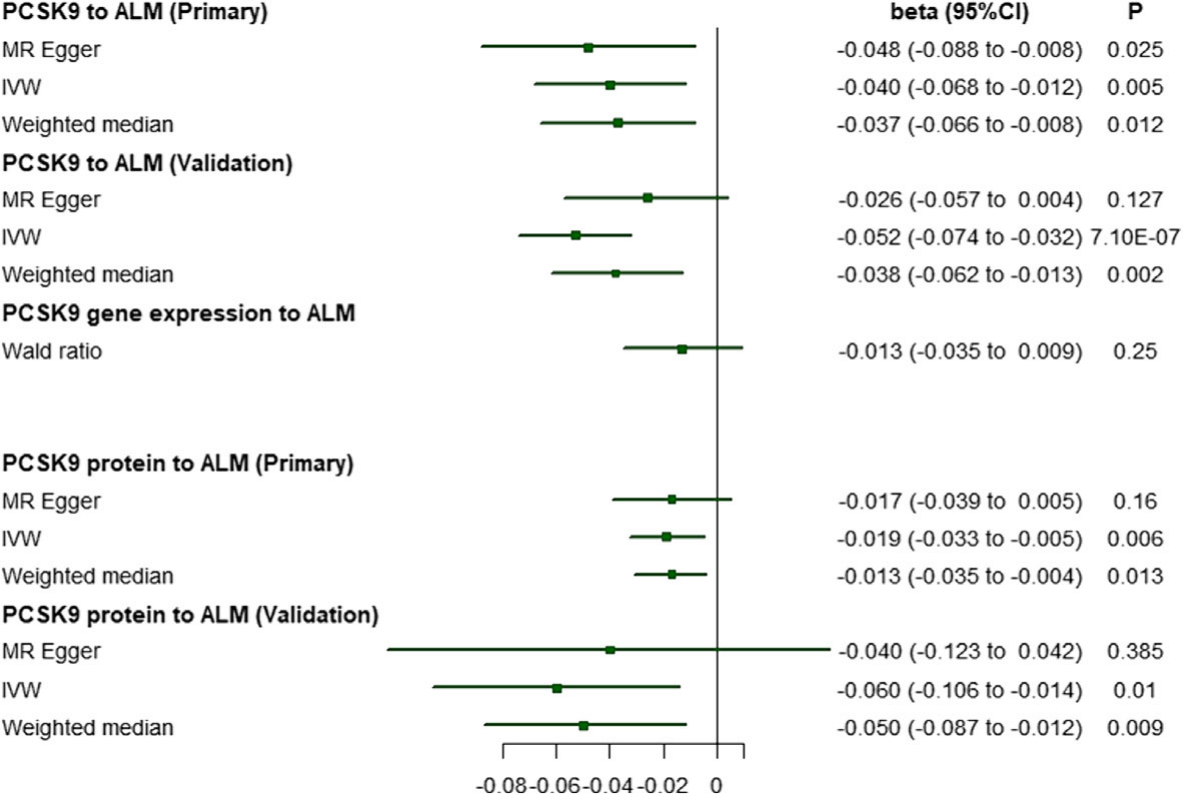
Drug‐target MR results of genetically proxied PCSK9 inhibition on ALM using genetic variants and cis‐QTLs instruments. ALM, appendicular lean mass; CI, confidence interval; PCSK9, proprotein convertase subtilisin/kexin type 9.

Based on the results from the IVW method, genetically proxied PCSK9 inhibition (due to genetic predisposition across 36 SNPs) was associated with reduced ALM (beta, −0.040; 95% CI, −0.068 to −0.012; *P =* 0.005; Figure 3). Sensitivity analyses using MR–Egger and the weighted median provided similar estimates in terms of direction and magnitude, and they were unlikely to have happened by chance alone (Figure 3). A secondary set of analyses using a set of 11 SNPs instead of 36 SNPs for PCSK9 yielded consistent results in terms of direction and magnitude (Figure 3). In addition, genetically proxied PCSK9 inhibition may increase the risk of LHGS as indicated by the EWGSOP criteria (odds ratio [OR], 1.09 per standard deviation inhibition in LDL; 95% CI, 1.01 to 1.18; *P =* 0.04), which was consistent with the FNIH criteria (OR, 1.10; 95% CI, 0.99 to 0.123; *P =* 0.065) in terms of direction, but the latter provided less precise estimates.

There was little statistical evidence of an association between HMGCR or NPC1L1 inhibition and sarcopenia risk (including ALM and LHGS) in either set of genetic instruments, and sensitivity MR analyses showed consistent estimates, with no statistical evidence of bias from horizontal pleiotropy.

### Genetically proxied inhibition in PCSK9 expression and sarcopenia

Table S11 presents the causal estimates between genetically proxied inhibition in circulating PCSK9 levels and patient outcomes. In line with the results of the PCSK9 drug‐target control MR analyses, a genetically proxied inhibition in the PCSK9 protein level was associated with a lower risk of CHD in two sets of PCSK9 cis‐pQTLs. Sensitivity MR analyses provided consistent results, while MR–Egger analysis provided less precise estimates. We did not identify evidence of directional pleiotropy (all MR–Egger intercepts *P* > 0.05). A one‐unit decrease in the normalized genetically predicted PCSK9 protein level was associated with a lower ALM (beta, −0.019; 95% CI, −0.033 to −0.005; *P =* 0.006; Figure 3), and the weighted median provided similar results in terms of direction and magnitude. MR–Egger analysis provided similar results in terms of direction, but the estimates were less precise. A secondary set of analyses using a set of seven SNPs yielded consistent results (Figure 3).

As expected, lower genetically predicted PCSK9 gene expression in the liver was associated with a lower risk of CHD [OR, 0.833; 95% CI, 0.744 to 0.931; *P =* 0.001]. A genetic proxied inhibition in PCSK9 gene expression in the liver may be associated with reduced ALM (beta, −0.013; 95% CI, −0.035 to 0.009; *P =* 0.25; Figure 3). This finding was consistent with the results of PCSK9 drug‐target MR analyses and PCSK9 protein MR analyses, but the magnitude was less precise. The detailed results were presented in the Table S12.

### Colocalization analysis

The posterior probability of colocalization between LDL and ALM in the PCSK9 gene region was 98% conditional on the presence of a causal variant for the outcome both in the primary analysis and validation analysis (Table S13). The full results of the colocalization analyses between LDL and ALM in the PCSK9 gene region are presented in the Table S13.

## Discussion

This MR study did not support the repurposing of lipid‐lowering drugs that inhibit HMGCR and NPC1L1 to delay or prevent sarcopenia risk. Our results suggest that exposure to PCSK9 inhibitors may predispose individuals to low muscle mass, although the magnitude of relative risk observed was small. The association between PCSK9 inhibition and ALM appeared to be independent of circulating LDL levels, as no association was observed between LDL and ALM overall.

We did not observe an association between LDL and ALM and did not identify support for the repurposing of lipid‐lowering drugs that inhibit HMGCR and NPC1L1 to delay or prevent sarcopenia risk. If the use of lipid‐lowering medications in general does not affect sarcopenia risk, this may signify the absence of a substantial role of dyslipidaemia in sarcopenia aetiology. Findings from many epidemiological studies have shown that higher LDL levels may be associated with an increased risk of sarcopenia,^26^, ^27^ and LDL cholesterol has been implicated in various processes that could contribute to sarcopenia, such as inflammation, oxidative stress, and impaired muscle protein synthesis. However, a study reported that there was no significant difference in LDL between people with sarcopenia and people without sarcopenia.^28^ These studies revealed a controversial relationship between dyslipidaemia and sarcopenia. To date, there is a lack of RCTs addressing the effect of therapeutic LDL reduction on sarcopenia risk. Taken together, these findings revealed no significant genetic associations for lowering LDL and no associations for the drug targets HMGCR and NPC1L1, suggesting that observational associations between higher LDL and sarcopenia risk may have been biased due to residual confounding.

Our results suggest that exposure to PCSK9 inhibitors might increase the risk of low muscle mass in individuals, although the observed relative risk was small in magnitude. Concerns about the potential adverse neurocognitive effects of PCSK9 inhibition have been raised based on a network meta‐analysis of RCTs.^29^ Taken together, our findings indicate that if the function of PCSK9 plays a role in determining the risk of sarcopenia, it likely occurs through pathways other than the regulation of peripheral LDL. PCSK9 plays a pivotal role in cholesterol metabolism by promoting the degradation of LDL receptors, thereby influencing cardiovascular disease risk.^30^ Elevated levels of circulating PCSK9 have been observed in individuals with diabetes, chronic kidney disease and psoriasis.^30^, ^31^, ^32^, ^33^ Low ALM or LHGS are commonly observed in patients with these diseases, leading to reasonable speculation about the potential involvement of PCSK9 in the pathogenesis of sarcopenia. In contrast to some findings from RCTs and systematic reviews of RCTs that have shown no significant difference in serious events such as myalgia between patients treated with PCSK9 inhibitors and control participants,^34^, ^35^ accumulating studies have detected a risk of musculoskeletal adverse events (MAEs) associated with PCSK9 inhibitors.^36^, ^37^, ^38^, ^39^ To date, limited data exist on the risk of sarcopenia associated with the use of PCSK9 inhibitors, and further careful evaluation of the long‐term safety of PCSK9 inhibitors is warranted. Additional mechanistic and more robust epidemiologic studies are needed to further explore this association.

This study has important clinical implications. This finding suggests the necessity of establishing a registry to monitor sarcopenia‐related outcomes in individuals who are inadvertently exposed to PCSK9 inhibitors to assess whether any signals are repeated. Integrating our findings into clinical practice involves developing personalized treatment plans that consider both lipid and muscle health. Potential strategies include stratifying patients by sarcopenia risk and tailoring lipid‐lowering therapy accordingly. Practically, the findings do not recommend any alteration to current recommendations regarding these drugs. However, clinicians should be acutely aware of the need for meticulous care for elderly patients receiving PCSK9 inhibitors, as these safety concerns could exacerbate existing disparities in cardiovascular care. Challenges include the need for longitudinal studies and clinical trials to validate these strategies and the consideration of individual patient characteristics such as age, sex, and co‐morbidities.

Investigating the association of lipid‐lowering drugs with sarcopenia risk using cohort designs would likely be limited by indication bias and reverse causation. The small number of patients prescribed PCSK9 inhibitors presents additional challenges. When IV assumptions are met, MR estimates the causal exposure–outcome association with less bias from unmeasured confounding. In our study, we developed genetic instruments to act as proxies for lipid‐lowering agents using three methods (i.e., GWAS, eQTLs, and pQTLs). MR analysis using cis instruments from QTLs is less likely to violate the assumption of no horizontal pleiotropy. Moreover, the causal association between PCSK9 inhibition and low ALM was consistently demonstrated by various sources of IVs and sensitivity tests in this study, improving causal inference and strengthening the validity of our genomic analytical model.

There are several limitations to consider. First, MR estimates capture the lifelong effects of genetic variants, whereas drugs are typically administered later in life. The subtle differences in PCSK9 levels through genetic variation cannot be directly compared with the effects of pharmacological inhibition, as the duration and timing of exposure differ. Therefore, cautious interpretation is warranted when assessing the size of these effects. Second, the study population consisted predominantly of individuals of European ancestry. The predominance of individuals of European ancestry in our study limits the generalizability of our findings. Future research should focus on diverse populations to validate these results and ensure their applicability across different ethnic groups. This includes conducting similar MR analyses in cohorts with varied ancestries and exploring potential genetic and environmental interactions. Third, as with all MR studies, IV assumptions are not empirically verifiable. It is possible that pleiotropy may bias the current estimates, although we performed sensitivity analyses using robust methods including weighted median and MR Egger approaches which did not identify the presence of directional pleiotropy. Finally, we were unable to evaluate potential off‐target effects and pathways of PCSK9 inhibition, including any effects occurring through mechanisms outside their respective lipid‐lowering pathways.

This MR study supported the link between decreased LDL via PCSK9 variants, decreased PCSK9 protein levels and decreased ALM, consistent with observational data showing an inverse relationship between PCSK9 and MAEs. Our results support current manufacturer recommendations to avoid the use of PCSK9 inhibitors among elderly people. Physicians caring for elderly patients who are being treated with PCSK9 inhibitors should exercise caution, although further clinical studies are needed to confirm these findings.

## Conflict of interest

None declared.

## Funding

Wan‐Qiang Lv was partially supported the National Natural Science Foundation of China (82301779), Zhejiang Provincial Natural Science Foundation of China (LQ24H250002), and Basic Research Project of Hangzhou Medical College (KYYB202201). Ruimin Sun was partially supported Basic Research Project of Hangzhou Medical College (KYYB202205). We thank the COVID‐19 Host Genetics Initiative and other consortium for making GWAS summary statistics publicly available. The summary GWASs data used in our study could be downloaded in the MR‐Base platform for researchers (www.mrbase.org/).

## Supporting information


**Table S1.** Genetic variants used to instrument LDL for the primary analysis
**Table S2.** Genetic variants used to instrument LDL for the validation analysis
**Table S3.** Detailed information of the enrolled drug targets identified from DrugBank and ChEMBL databases
**Table S4.** Genetic variants used to instrument each lipid lowering drug target for the primary analysis
**Table S5.** Genetic variants used to instrument each lipid lowering drug target for the validation analysis
**Table S6.** Genetic variants selected from their association with pQTL to proxy PCSK9 inhibition in the primary analysis
**Table S7.** Genetic variants selected from their association with pQTL to proxy PCSK9 inhibition in the validation analysis
**Table S8.** Genetic variants selected from their association with eQTL to proxy PCSK9 inhibition in the liver.
**Table S9.** Associations between genetically proxied reduction in LDL overall and outcomes (appendicular lean mass, low hand grip strength using EWGSOP and FNIH criteria and coronary heart disease)
**Table S10.** Associations between genetically proxied drug targets and outcomes (appendicular lean mass, low hand grip strength using EWGSOP and FNIH criteria and coronary heart disease)
**Table S11.** Associations between genetically proxied reduction in circulating PCSK9 level and ALM.
**Table S12.** Associations between genetically proxied reduction in liver PCSK9 gene expression and ALM.
**Table S13.** The posterior probability of colocalization via Bayesian tests between LDL and ALM in the PCSK9 gene region.
